# Body Composition and Serum Concentration of Thyroid Hormones in Euthyroid Men and Women from General Population

**DOI:** 10.3390/jcm11082118

**Published:** 2022-04-11

**Authors:** Agnieszka Adamska, Andrzej Raczkowski, Zofia Stachurska, Marcin Kondraciuk, Adam Jacek Krętowski, Marcin Adamski, Irina Kowalska, Karol Adam Kamiński

**Affiliations:** 1 Department of Endocrinology, Diabetology and Internal Medicine, Medical University of Białystok, 15-089 Białystok, Poland; adamkretowski@wp.pl; 2 Department of Population Medicine and Civilization Diseases Prevention, Medical University of Bialystok, 15-089 Białystok, Poland; andrzej.raczkowski@umb.edu.pl (A.R.); zofia.stachurska@umb.edu.pl (Z.S.); marcin.kondraciuk@umb.edu.pl (M.K.); karol.kaminski@umb.edu.pl (K.A.K.); 3 Clinical Research Center, Medical University of Bialystok, 15-089 Białystok, Poland; 4 Faculty of Computer Science, Bialystok University of Technology, 15-351 Bialystok, Poland; mm.adamski@wp.pl; 5 Department of Internal Medicine and Metabolic Diseases, Medical University of Białystok, 15-089 Białystok, Poland; irina.kowalska@umb.edu.pl

**Keywords:** body composition, thyroid hormones, cohort study

## Abstract

Body composition, especially an increased amount of fat mass and decreased lean body mass, is connected with metabolic complications. Thyroid hormones can influence body composition pattern. To date, scarce data defining the relationships between thyroid hormones and parameters of body composition using dual-energy X-ray absorptiometry (DXA), especially in cohort studies, are available. Therefore, the aim of the present study was to investigate the relationships among serum concentrations of (thyroid-stimulating hormone (TSH), thyroid hormones, and distribution of fat tissue assessed using the DXA method in a euthyroid cohort from the Białystok PLUS study. We examined 582 euthyroid subjects who were divided into lean (body mass index (BMI) < 25 kg/m^2^) and overweight/obese (BMI ≥ 25 kg/m^2^) (84 lean men, 182 overweight/obese men, 160 lean women, and 156 overweight/obese women). Serum concentrations of TSH, free T3 (fT3), and free T4 (fT4) were assessed, and DXA was performed. We observed lower serum levels of fT4 (*p* = 0.03) and higher serum levels of fT3 (*p* = 0.04) in overweight/obese vs. lean men, whereas serum levels of TSH did not differ between these groups (*p* = 0.38). In lean men, we only observed a relationship between serum levels of TSH and visceral adipose tissue (VAT) (*r* = −0.24, *p* = 0.02). In overweight/obese men, we found that serum levels of fT3 were positively connected with total fat mass (*r* = 0.16, *p* = 0.02), android fat mass (*r* = 0.15, *p* = 0.03), and gynoid fat mass (*r* = 0.17, *p* = 0.01), but not with VAT (*r* = 0.03, *p* = 0.63). We did not observe differences in serum levels of TSH, fT3, and fT4 between lean and overweight/obese women. Additionally, we did not notice relationships between serum levels of thyroid hormones and fat in different regions estimated by DXA in lean and overweight/obese women (all *p* > 0.05). We concluded that the serum concentration of TSH is connected with VAT in lean men, whereas, in overweight/obese men, higher fT3 is connected with an increased fat amount. These associations are absent in women.

## 1. Introduction

The growing prevalence of overweight and obesity is becoming an increasingly important health problem all over the world. It is well established that overweight and obesity are risk factors for diabetes, hypertension, cardiovascular diseases, and some types of cancer [1]. In particular, body composition, especially increased amount of fat mass and decreased lean body mass, is connected with many metabolic complications [1]. Additionally, it has been demonstrated that visceral adipose tissue (VAT) is metabolically more active than subcutaneous adipose tissue, and that an increased amount of VAT is associated with higher risk of metabolic disturbances, e.g., hypertension, dyslipidemia, insulin resistance, and type 2 diabetes [2]. Moreover, the wellbeing of women is mainly associated with the distribution of adipose tissue [3].

Thyroid hormones play an important role in determining body mass, as well as body composition [4]. There is evidence that thyroid hormones are connected with cardiovascular risk factors and events not only in hyper- or hypothyroidism, but also within the reference range [4,5]. However, in the euthyroid range of thyroid status, it is unclear whether changes in markers of thyroid function precede changes in weight or vice versa, and obtained results are not consistent [6,7,8]. In subjects without thyroid disease, some studies have reported correlations between thyroid-stimulating hormone (TSH), free T4 (fT4), and free T3 (fT3) in the normal reference range and body mass, body composition, weight gain, or energy expenditure. However, the correlations were in different directions, while other studies failed to find relationships between thyroid function and these outcomes [9,10,11]. It has been shown that body mass index (BMI) and waist circumference are positively associated with levels of serum TSH and fT3 among euthyroid adults, and that every one-quartile increase in BMI is associated with an increase in serum fT3 and TSH levels in euthyroid men and women [9]. In the Framingham Offspring Study (*n* = 2407), it was shown that an increase in serum concentration of TSH within the reference range could be connected with weight gain [7]. In another study, in obese euthyroid subjects, TSH was positively associated with the percentage of body fat measured by electrical bioimpedance [12], whereas it was demonstrated that decreases in TSH were associated with weight loss [13]. However, the obtained results are inconsistent; therefore, this issue needs more explanation [6,7,8]. Accordingly, in the euthyroid range of thyroid status, it is unclear whether changes in markers of thyroid function precede changes in weight, or vice versa. To date, several cohort studies defining the parameters of body composition using dual-energy X-ray absorptiometry (DXA) method have been performed [14,15]. With this method, the assessment of fat and lean mass in the android and gynoid region and of VAT mass can be performed. Nevertheless, cohort studies focused on thyroid function and body composition are limited. 

In face of the fact that the impact of the serum concentration of thyroid hormones on fat distribution is not clear, we assessed the relationship between serum concentration of thyroid hormones and body composition using the DXA method in a euthyroid cohort from general population—Białystok PLUS study (Polish Longitudinal University Study (Bialystok+)).

## 2. Materials and Methods

### 2.1. Subjects

The study was performed in 2017–2020 in a representative sample of area residents at the age of 20 to 80. According to the 2017 Central Statistical Office data, the number of residents in Bialystok was 297,300. Randomly selected residents (994) from the mayor’s office database were invited to participate in the study. All the subjects participating in the study were Caucasians. Subjects were excluded for the intake of any medication that might affect thyroid status or a history of thyroid diseases, including diagnosed overt hyperthyroidism or hypothyroidism, thyroid cancer, or thyroid surgery. Finally, 582 euthyroid individuals including 266 males and 316 females were considered eligible for this study. Euthyroid was defined as a serum level of TSH, fT3 and fT4 within the normal range. 

### 2.2. Study Protocol

The subjects’ medical records were obtained from the questionnaires filled at the time of entry. All study participants underwent physical examination and laboratory assessment. BMI was calculated as body weight in kilograms divided by height in meters squared (kg/m^2^). Waist circumference was measured in the standing position, at the smallest circumference between the rib cage and the iliac crest. Hip circumference was measured in the standing position. Waist–hip ratio (WHR) was calculated as the waist circumference (cm) divided by hip circumference (cm). Normal weight was defined as BMI < 25 kg/m^2^, and overweight/obesity was defined as BMI ≥ 25 kg/m^2^. 

### 2.3. Biochemical Analyses

Peripheral fasting blood samples were collected in the morning on a visit day. Blood samples were stored at −80 °C until the analyses of TSH, fT3, fT4, and serum insulin concentrations. Serum TSH concentration was estimated using the electrochemiluminescence method (ECLIA) (Cobas e411, ROCHE Diagnostic Ltd., Rotkreuz, Switzerland) (sensitivity: 0.005 µIU/mL; intra-assay CV: 3.95%; inter-assay CV: 1.45%). Serum fT3 (sensitivity: 1.5 pmol/L; intra-assay CV: 2.75%; inter-assay CV: 1.65%), serum fT4 (sensitivity: 0.8 pg/mL; intra-assay CV: 3.2%, inter-assay CV: 1.85%), and serum insulin concentrations (sensitivity: 0.2 µU/mL, intra-assay CV: 3.60%; inter-assay CV: 1.25%) were estimated using the electrochemiluminescence method (ECLIA) (Cobas e411, ROCHE Diagnostic Ltd., Rotkreuz, Switzerland). Plasma glucose levels and concentrations of serum total cholesterol, high-density lipoprotein cholesterol (HDLcholesterol), and triglycerides were measured immediately after the blood was collected. Plasma glucose levels were assessed by the hexokinase method, and concentrations of serum total cholesterol, HDL-cholesterol, and triglycerides were measured using the enzymatic colorimetric method (Cobas c111, Roche Diagnostic Ltd., Rotkreuz, Switzerland). Serum low-density lipoprotein cholesterol (LDL cholesterol) concentration was calculated using Friedewald’s formula. 

### 2.4. Body Composition Analysis

Body composition analysis was conducted using DXA (GE Healthcare, Chicago, IL, USA, Lunar iDXA) by qualified physicians at the Clinical Research Center, Medical University of Bialystok. The equipment was calibrated before every examination. The patients were positioned on the examination table in a supine position, with their feet secured together with an adjustable strap and hands lying flat adjacent to the sides of the body. Each examination took approximately 8 min. With this method, body composition consisting of body fat (kg) and lean mass was estimated. For each region of the whole body (head, trunk, arms, and legs), lean and fat body mass was determined. On the basis of the scans, CoreScan software estimated VAT within the android region. DXA assessed fat mass with a precision and coefficient of variation of 2.0% and 8.0%, respectively. 

### 2.5. Calculations

The homeostasis model assessment of insulin resistance (HOMA-IR) was calculated as fasting insulin (μIU/mL) × fasting plasma glucose (mmol/L)/22.5 [16].

### 2.6. Statistical Analysis

The statistical analyses were performed using Statistica 13.3 package (Statsoft, Cracow, Poland). The variables were tested for normal distribution using the Shapiro–Wilk test. Due to non-normal distribution of the data, nonparametric tests were applied, and all values were expressed as the median and interquartile range. The Spearman test was used for correlation analysis. A *p*-value <0.05 was considered statistically significant.

## 3. Results

The clinical and biochemical characteristics of the studied women are presented in Table 1. Lean women were younger in comparison to overweight/obese women (*p* = 0.01). In accordance with the assumption of the study, we observed differences in BMI, WHR, HOMA-IR, serum lipids, and fat and lean mass between lean and overweight/obese women (all *p* < 0.05). However, we did not notice differences among serum levels of TSH, fT3, and fT4 between lean and overweight/obese women (all *p* > 0.05) (Table 1). 

The clinical and biochemical characteristics of the studied men are presented in Table 2. Lean men were younger in comparison to overweight/obese men (*p* = 0.02). In accordance with the assumption of the study, we observed differences in BMI, WHR, HOMA-IR, serum HDL cholesterol, LDL cholesterol, triglycerides, and fat and lean mass between lean and overweight/obese men (all *p* < 0.05). We did not notice differences between serum levels of TSH between lean and overweight/obese men (all *p* = 0.38), although we noticed lower serum levels of fT4 (*p* = 0.03) and higher serum levels of fT3 (*p* = 0.04) in overweight/obese vs. lean men (Table 2). 

We did not notice relationships of serum levels of TSH, fT4, and fT3 with total fat mass, gynoid fat mass, android fat mass, or VAT in lean and overweight/obese women (all *p* > 0.05) (Table 3). 

We did not notice a relationship between HOMA-IR and serum levels of TSH in overweight/obese (*r* = 0.06, *p* = 0.4) or in lean women (*r* = −0.05, *p* = 0.5). We also did not observe a relationship between HOMA-IR and serum concentration of fT3 and fT4 in overweight/obese women (*r* = 0.08, *p* = 0.3; *r* = −0.08, *p* = 0.29, respectively) or in lean women (*r* = 0.06, *p* = 0.4; *r* = 0.24, *p* = 0.3, respectively).

**Figure 1:**
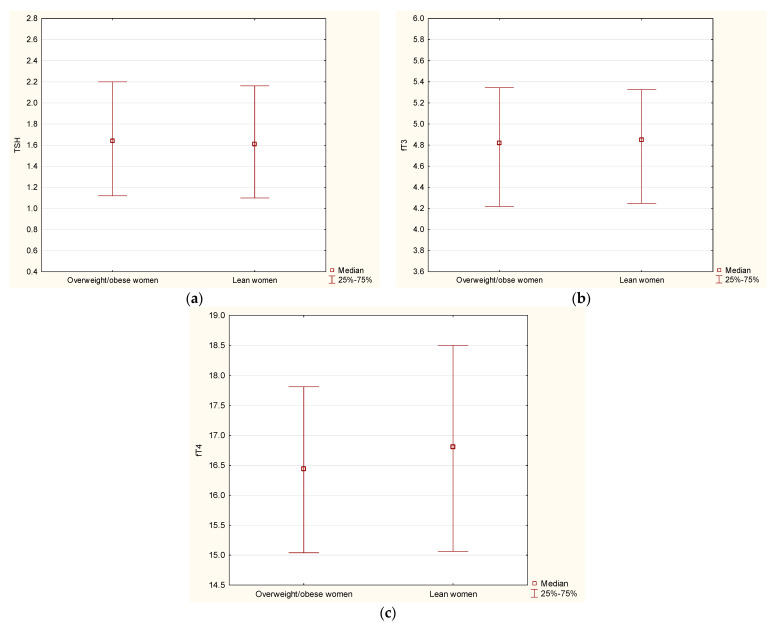
Serum levels of TSH (**a**), fT3 (**b**), and fT4 (**c**) in overweight/obese women (*n* = 149) and lean (*n* = 160) women.

We performed an additional analysis in the subgroup of women with similar age but different BMI. Similarly, as shown previously, we did not observe differences in serum levels of TSH, fT3, and fT4 between overweight/obese women (*n* = 149) and lean women (*n* = 160) (all *p* > 0.05) (Figure 1).

Additionally, we did not notice relationships between serum levels of TSH or thyroid hormones and fat in different regions estimated by DXA in lean and overweight/obese women (all *p* > 0.05).

In lean men, we did not notice a relationship between serum levels of fT3 and fT4 and the parameters estimated by DXA (all *p* > 0.05), although we observed a relationship between serum levels of TSH and VAT (*r* = −0.24, *p* = 0.02). In overweight/obese men, we found that serum levels of fT3 were positively connected with total fat mass (*r* = 0.16, *p* = 0.02), android fat mass (*r* = 0.15, *p* = 0.03) and gynoid fat mass (*r* = 0.17, *p* = 0.01), but not with VAT (*r* = 0.03, *p* = 0.63) (Table 4). 

Accordingly, we noticed a relationship between HOMA-IR and serum levels of fT3 in overweight/obese men (*r* = 0.25, *p* < 0.01) but not in lean men (*r* = 0.13, *p* = 0.2). Moreover, we observed a negative relationship between HOMA-IR and serum concentration of fT4 in overweight/obese men (*r* = −0.23, *p* < 0.01) but not in lean men (*r* = 0.09, *p* = 0.4).

**Figure 2:**
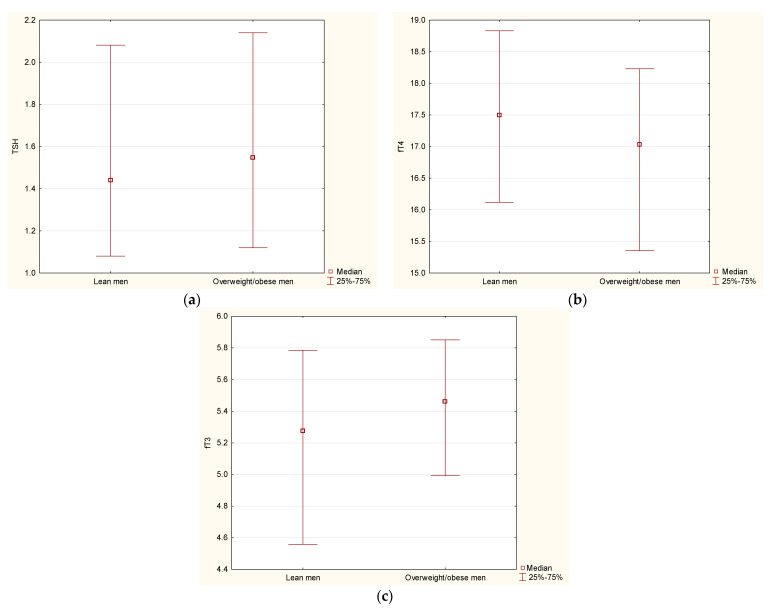
Serum levels of TSH (**a**), fT4 (**b**), and fT3 (**c**) in lean (*n* = 84) vs. overweight/obese men (*n* = 175).

We performed additional analyses in the subgroup of men with similar age but different BMI. Similarly, as shown previously, in the subgroup analysis, we observed lower serum levels of fT4 (*p* = 0.04) and higher serum levels of fT3 (*p* = 0.04) in overweight/obese (*n* = 175) vs. lean men (*n* = 84), whereas serum levels of TSH did not differ between these groups (*p* = 0.38) (Figure 2).

In lean men, we observed a relationship between serum levels of TSH and VAT (*r* = −0.24, *p* = 0.02). In overweight/obese men, we found that serum levels of fT3 were positively connected with total fat mass (*r* = 0.18, *p* = 0.02), android fat mass (*r* = 0.16, *p* = 0.02), and gynoid fat mass (*r* = 0.18, *p* = 0.01), but not with VAT (*r* = 0.05, *p* = 0.5).

## 4. Discussion

In the present investigation of a large, representative sample from the Białystok PLUS study, we observed lower serum levels of fT4 and higher serum levels of fT3 in overweight/obese vs. lean men. We also found a negative relationship between serum levels of TSH and VAT in lean men, whereas, in overweight/obese men, we observed that serum levels of fT3 were positively connected with total, android, and gynoid fat mass, but not with VAT. Interestingly, we observed a positive relationship between HOMA-IR and serum levels of fT3 in overweight/obese men and a negative relationship between HOMA-IR and serum concentration of fT4 in overweight/obese men. However, we did not notice relationships of serum levels of TSH, fT3, and fT4 with total, gynoid, and android fat mass and VAT in lean and overweight/obese women. In a previous study it was shown that estrogens induced an increase in total thyroxine (TT4) and total triiodothyronine (TT3) values [17], whereas deficiency of these hormones promotes metabolic dysfunction predisposing to obesity in women [18]. Therefore, this may explain the correlations between thyroid hormones and fat mass and HOMA-IR observed in our study, present in men but not in women.

In the present study, we did not observe differences between serum levels of TSH, fT3, and fT4 in lean vs. overweight/obese women, although, in men, serum concentration of fT4 was lower, while that of fT3 was higher in overweight/obese vs. lean participants. These findings are consistent with the reports by Ren et al., who showed elevated serum levels of fT3 in overweight and obese subjects compared with normal-weight subjects [19]. Additionally, other studies found an elevated serum level of fT3 (although in the upper limit of the normal range) in overweight/obese subjects in comparison to normal-weight subjects [20]. However, in another study, the authors did not observe differences in serum levels of fT4 between subjects with BMI in the reference norm and with overweight/obesity [19], which may be attributed to the fact that women and men were examined together, whereas, in our study, we analyzed men and women separately. In the euthyroid state, it is not clear if changes in thyroid hormones precede changes in weight, or changes in weight impact the pituitary–thyroid axis. It can be speculated that normal serum levels of T3 seem to be important to maintain body weight by attempting to regain a zero energy balance [10]. Therefore, our results could indicate that, in overweight/obese men, there is increased conversion of T4 to T3. These findings may be explained by increased serum leptin levels in the overweight/obese state [21] and its impact on an increased activity of type 1 deiodinase, which is responsible for the conversion of T4 to T3 [22]. Another explanation of our findings could be connected with the insulin resistance presented in obesity [23] and the stimulating effect of insulin on type 2 deiodinase activity, as shown in rat brown adipocytes [24]. As mentioned previously, we noticed a positive relationship between HOMA- IR and serum levels of fT3 in overweight/obese men and a negative connection between HOMA-IR and serum concentration of fT4 in overweight/obese men. Therefore, increased activity of specific deiodinases in obesity could be an adaptive process to stop further weight gain by increasing energy expenditure to avoid the accumulation of fat [10]. Consequently, in our study, we observed positive relationships between serum levels of fT3 and parameters of body fat composition, e.g., total, android, and gynoid fat mass. On the basis of this observation, we can assume that an increase in fat amount is connected with increased serum levels of fT3 in overweight/obese men. However, we did not observe these relationships in overweight/obese women. It was shown that single-nucleotide polymorphism Thr92Ala in the coding region type 2 iodothyronine deiodinase, encoded by DIO2, is associated with insulin resistance [25]. In our study, we did not notice a relationship between HOMA-IR and serum levels of fT4 and fT3 in overweight/obese women, as observed in overweight/obese men. Therefore, we can speculate that, in women with higher insulin resistance, differences in serum levels of thyroid hormones could probably be observed. On the other hand, it is possible that our sample size is still too small for a potential association. In the previous study conducted by Ren et al., it was shown that serum concentration of fT3 was associated with fat mass estimated by bioelectrical impedance in euthyroid subjects [19]. Similar to our results observed in overweight/obese men, Samuels et al. showed that serum fT3 levels were directly correlated with body fat mass estimated using DXA in levothyroxine (L-T4)-treated men and women [26]. Additionally, the authors observed a relationship between serum levels of fT3 and VAT. This relationship is difficult to explain because T3 stimulates lipolysis and decreases body fat in animal models [5]. In our study, we did not observe relationships between fT3 and VAT in men and women, although we excluded subjects treated with L-T4; thus, it is difficult to compare the obtained results. 

The presented results do not indicate whether the disturbances in serum concentrations of thyroid hormones in overweight/obese vs. lean men contribute to obesity or are its consequence per se. In our study, we showed that, in euthyroid men, an increase in fat mass (total, android, gynoid) is associated with disturbances in the concentrations of thyroid hormones. Therefore, as our results indicate that excess fat mass may contribute to changes in thyroid function in men, even in the euthyroid state, weight reduction may also be important in this group to achieve balance in thyroid function. On the other hand, disturbances in thyroid hormones may contribute to metabolic complications of excess body fat and associated insulin resistance in overweight/obese men. In order to clarify this issue, we conducted the Białystok PLUS study assessing individual parameters after 5 years, which may help in answering the question posed. As mentioned in Section 1, VAT is metabolically more active than subcutaneous adipose tissue, and an increased amount of VAT is associated with a higher risk of metabolic disturbances [2]. Additionally, it has been shown that VAT could be a predictor of TSH [27]. Obviously, in our study, overweight/obese women and men had a higher content of VAT vs. lean women and men, but this was not related to the serum concentration of TSH. Therefore, in the euthyroid state, thyroid hormones are not connected with higher VAT mass in overweight/obese women and men.

In previous studies, it was demonstrated that serum levels of TSH are slightly increased in obese subjects as compared to normal-weight humans as a consequence of obesity [10]. In our study, we did not confirm this observation. This could be connected with the characteristics of the studied groups because, in most studies, the mean BMI of subjects was higher than in our study. Additionally, in most studies, subjects with thyroid disease were not excluded. However, we observed a negative relationship between serum levels of TSH and VAT in lean men but not in overweight/obese subjects. In the study conducted by Muscogiuri et al., using computed tomography scans to determine VAT, the authors showed that VAT was the most powerful predictor of TSH [27]. Contrary to these findings, in the study conducted on a sample of 1021 women and 956 men in the population-based Study of Health in Pomerania Trend (SHIPTrend), no relationship was observed between serum levels of TSH and VAT [28], although the authors did not stratify the groups by BMI and gender and did not exclude subjects with thyroid disease, as we did. On the other hand, they assessed VAT using gold-standard methods, e.g., whole-body magnetic resonance imaging. On the basis of our results, we can speculate that serum levels of TSH in lean men may be connected with an increase in visceral fat in the future.

In the present study, we did not notice relationships of serum levels of TSH, fT3, and fT4 with total, gynoid, and android fat mass and VAT in lean and overweight/obese women. This is partly consistent with the results obtained by Witte et al. [28]. However, an Italian study showed a relationship between serum levels of TSH and VAT [27]. One explanation of these results could be connected with the fact that, in the previous study, the sample size was small, and the characteristics of the population differed from our general population sample. The other explanation could be connected with the fact that a positive association may have been overlooked in specific subgroups due to insufficient statistical power. On the other hand, it is possible that our sample size is still too small for a potential association.

The main limitation of the present study is the fact that our results did not allow us to identify the definite cause-and-effect relationship. Additionally, in some of the results, the level of statistical significance was close to 0.05; therefore, the results need to be corroborated by additional studies. The conducted study was an observational one, and, in order to prove the cause-and-effect relationship, it would be necessary to perform a long-term follow-up. However, longitudinal data will be available from the follow-up of the Białystok PLUS study in five years, and we will be able to verify our results. Additionally, further methodological research should examine the extent of the representativeness of this research. The study population was between 20 and 80 years, and the comorbidities of the patients were not taken into account, which could have biased the results, as low fT3 is frequently associated with chronic illness. However, we examined subjects without thyroid diseases and strictly euthyroid subjects with TSH, fT3, and fT4 all in the normal range. The major strength of this study was its large size, which allowed for a relatively precise evaluation of the associations of interest. Additionally, DXA was performed in all subjects.

## 5. Conclusions

We concluded that the serum concentration of TSH is connected with VAT in lean men, whereas, in overweight/obese men, higher fT3 is connected with increased fat amount. These associations were absent in women.

## Figures and Tables

**Table 1:** Clinical and biochemical characteristics and body composition estimated with DXA method in lean and overweight/obese women.

	Lean Women (*n* = 160)	Overweight/Obese Women (*n* = 156)	*p*
Age (years)	44.5 (36.5–59)	52 (42–60)	0.01
BMI (kg/m^2^)	22.3 (20.4–23.4)	28.9 (26.8–32.3)	<0.01
WHR	0.78 (0.75–0.83)	0.83 (0.8–0.88)	<0.01
Total cholesterol (mg/dL)	187 (161–210)	193 (173–222)	0.04
HDL cholesterol (mg/dL)	72 (61–80)	61 (53–71)	<0.01
LDL cholesterol (mg/dL)	115 (95–139)	128 (104–153)	<0.01
Triglycerides (mg/dL)	74 (58–97)	102 (72–139)	<0.01
TSH (uIU/mL)	1.61 (1.1–2.16)	1.63 (1.09–2.21)	0.89
fT4 (pmol/L)	16.8 (15.1–18.5)	16.6 (15.1–17.9)	0.19
fT3 (pg/mL)	4.85 (4.25–5.33)	4.84 (4.24–5.35)	0.92
Total fat mass (kg)	19.5 (16.2–21.6)	31.3 (27.8–37.7)	<0.01
Gynoid fat mass (kg)	3.4 (2.9–3.9)	5.2 (4.6–6.1)	<0.01
Android fat mass (kg)	1.3 (0.9–1.7)	2.8 (2.3–3.5)	<0.01
Total lean mass (kg)	38.5 (36.2–41.2)	43 (41–47)	<0.01
Android lean mass (kg)	2.7 (2.5–2.9)	3.0 (2.8–3.3)	<0.01
Gynoid lean mass (kg)	3.4 (3.0–3.9)	6.5 (6.1–7.1)	<0.01
VAT mass (g)	302 (182–500)	1187 (703–1594)	<0.01
HOMA-IR	1.74 (1.39–2.34)	2.96 (2.17–4.02)	<0.01

Values are expressed as the median (interquartile range). Abbreviations: HDL cholesterol, high-density lipoprotein cholesterol; LDL cholesterol, low-density lipoprotein cholesterol; BMI, body mass index; WHR, waist-to-hip ratio; TSH, thyroid-stimulating hormone; fT4, free T4; fT3, free T3; DXA, dual-energy X-ray absorptiometry; VAT, visceral adipose tissue; HOMA-IR, homeostasis model assessment of insulin resistance.

**Table 2:** Clinical and biochemical characteristics and body composition estimated with DXA method in lean and overweight/obese men.

	Lean Men (*n* = 84)	Overweight/Obese Men (*n* = 182)	*p*
Age (years)	38.5 (30–57)	45.5 (36–59)	0.02
BMI (kg/m^2^)	22.9 (21.6–24.2)	28.7 (26.6–30.9)	<0.01
WHR	0.88 (0.85–0.92)	0.96 (0.92–1)	<0.01
Total cholesterol (mg/dL)	180 (154–206)	188 (167–218)	0.05
HDL cholesterol (mg/dL)	59 (52–69)	49 (42–59)	<0.01
LDL cholesterol (mg/dL)	115 (92–141)	128 (108–149)	<0.01
Triglycerides (mg/dL)	86 (58–100)	118 (80–166)	<0.01
TSH (uIU/mL)	1.44 (1.08–2.08)	1.54 (1.12–2.13)	0.38
fT4 (pmol/L)	17.49 (16.12–18.83)	16.98 (15.35–18.21)	0.03
fT3 (pg/mL)	5.28 (4.56–5.79)	5.42 (4.98–5.82)	0.04
Total fat mass (kg)	16.1 (12.4–19.4)	27.9 (23.4–33.1)	<0.01
Gynoid fat mass (kg)	2.4 (1.9–2.9)	3.8 (3.1–4.5)	<0.01
Android fat mass (kg)	1.2 (0.8–1.8)	3.1 (2.3–3.9)	<0.01
Total lean mass (kg)	52.5 (49–58.9)	60.5 (56–65.2)	<0.01
Android lean mass (kg)	3.6 (3.3–3.9)	4.0 (3.8–4.4)	<0.01
Gynoid lean mass (kg)	7.8 (7.2–8.6)	9.0 (8.3–9.8)	<0.01
VAT mass (g)	619 (330–1024)	1922 (1314–2646)	<0.01
HOMA-IR	1.76 (1.29–2.27)	3.33 (2.22–5.04)	<0.01

Values are expressed as the median (interquartile range). Abbreviations: HDL cholesterol, high-density lipoprotein cholesterol; LDL cholesterol, low-density lipoprotein cholesterol; BMI, body mass index; WHR, waist-to-hip ratio; TSH, thyroid-stimulating hormone; fT4, free T4; fT3, free T3; DXA, dual-energy X-ray absorptiometry; VAT, visceral adipose tissue; HOMA-IR, homeostasis model assessment of insulin resistance.

**Table 3:** Relationships between serum TSH, fT4, and fT3 concentrations and body composition parameters estimated with DXA in lean and overweight/obese women.

	Lean Women (*n* = 160)			Overweight/Obese Women (*n* = 156)		
	TSH	fT3	fT4	TSH	fT4	fT3
Total fat mass (kg)	*r* = −0.01, *p* = 0.8	*r* = −0.11, *p* = 0.16	*r* = 0.1, *p* = 0.19	*r* = 0.04, *p* = 0.5	*r* = −0.09, *p* = 0.22	*r* = −0.04, *p* = 0.54
Gynoid fat mass (kg)	*r* = −0.02, *p* = 0.7	*r* = −0.09, *p* = 0.22	*r* = 0.03, *p* = 0.62	*r* = 0.05, *p* = 0.5	*r* = −0.12, *p* = 0.11	*r* = −0.02, *p* = 0.77
Android fat mass (kg)	*r* = 0.01, *p* = 0.8	*r* = −0.08, *p* = 0.28	*r* = 0.11, *p* = 0.14	*r* = −0.02, *p* = 0.7	*r* = −0.05, *p* = 0.50	*r* = −0.08, *p* = 0.29
VAT mass (g)	*r* = −0.03, *p* = 0.6	*r* < 0.01, *p* = 0.92	*r* = 0.1, *p* = 0.19	*r* = −0.07, *p* = 0.3	*r* < −0.01, *p* = 0.96	*r* = 0.01, *p* = 0.83

Abbreviations: DXA, dual-energy X-ray absorptiometry; fT4, free T4; fT3, free T3; TSH, thyroid-stimulating hormone; VAT, visceral adipose tissue.

**Table 4:** Relationships between serum TSH and fT3 concentrations and body composition parameters estimated with DXA in lean and overweight/obese men.

	Lean Men (*n* = 84)			Overweight/Obese Men (*n* = 182)		
	TSH	fT3	fT4	TSH	fT3	fT4
Total fat mass (kg)	*r* = −0.09, *p* = 0.37	*r* = 0.01, *p* = 0.88	*r* = 0.09, *p* = 0.3	*r* = 0.05, *p* = 0.42	*r* = 0.16, *p* = 0.02	*r* = −0.04, *p* = 0.5
Gynoid fat mass (kg)	*r* = 0.01, *p* = 0.91	*r* < 0.01, *p* = 0.97	*r* = 0.2, *p* = 0.05	*r* = 0.12, *p* = 0.08	*r* = 0.17, *p* = 0.01	*r* = −0.04, *p* = 0.5
Android fat mass (kg)	*r* = −0.20, *p* = 0.06	*r* = 0.02, *p* = 0.85	*r* = 0.07, *p* = 0.4	*r* = 0.02, *p* = 0.70	*r* = 0.15, *p* = 0.03	*r* = −0.05, *p* = 0.4
VAT mass (g)	*r* = −0.24, *p* = 0.02	*r* < −0.01, *p* = 0.98	*r* = 0.03, *p* = 0.7	*r* = −0.08, *p* = 0.26	*r* = 0.03, *p* = 0.63	*r* = −0.09, *p* = 0.2

Abbreviations: DXA, dual-energy X-ray absorptiometry; TSH, thyroid-stimulating hormone; fT3, free T3; fT4, free T4; VAT, visceral adipose tissue.

## Data Availability

Data are available on request.
